# Glutamate metabotropic receptor 4 (GRM4) inhibits cell proliferation, migration and invasion in breast cancer and is regulated by miR-328-3p and miR-370-3p

**DOI:** 10.1186/s12885-019-6068-4

**Published:** 2019-09-06

**Authors:** Bin Xiao, Daxiang Chen, Quan Zhou, Jianfeng Hang, Weiyun Zhang, Zhenzhan Kuang, Zhaohui Sun, Linhai Li

**Affiliations:** 1 Department of Laboratory Medicine, General Hospital of Southern Theatre Command of PLA, Guangzhou, 510010 China; 2 Department of Laboratory Medicine, Dermatology Hospital, Southern Medical University, Guangzhou, China; 3 Department of Laboratory Medicine, Guangdong Provincial Dermatology Hospital, Guangzhou, China

**Keywords:** GRM4, Breast cancer, Proliferation, miR-328-3p, miR-370-3p

## Abstract

**Background:**

Glutamate metabotropic receptors (GRM) play a variety of roles in neuronal cells. However, their clinical significance and biological functions in breast cancer remain unknown.

**Methods:**

RNA sequencing data of breast cancer was obtained from the TCGA dataset (v2) and mined for the expression profiles of GRM family according to cancer subtypes. mRNA expression of GRM family in breast cancer tissues and para-cancerous tissue samples as well as breast cancer cell lines were measured by qPCR. The effects of over- and under-expression of GRM4 on cell capabilities to survive, migrate and invade were determined by colony formation, transwell migration and invasion assays. To explore the upstream regulation pattern of GRM4, miRNAs that target GRM4 were predicted and validated by dual luciferase reporter assay. In addition, the mRNA and protein expression of GRM4 regulated by these miRNAs were further measured by qPCR and western blot assay.

**Results:**

GRM4 was the only GRM member that expressed in breast cancer tissues. Ectopic expression of GRM4 was correlated with better prognosis of breast cancer patients. Overexpression of GRM4 could significantly inhibit cell proliferation, migration and invasion capacity in MDA-MB-231, while knockdown of GRM4 could promote these processes. miR-328-3p and miR-370-3p were predicted to regulate the expression of GRM4 and dual luciferase reporter assay demonstrated that miR-328-3p and miR-370-3p directly bound to the 3′ UTR of GRM4 and mutations on the binding regions on GRM4 significantly decreased the luciferase activity. qPCR demonstrated that expression of miR-328-3p and miR-370-3p was significantly decreased in breast cancer tissues and cells compared with that in control samples. However, there were no correlations between the expression of miR-328-3p and GRM4, as well as the expression of miR-370-3p and GRM4. Moreover, overexpression of miR-328-3p and miR-370-3p counteracted the inhibitory effect of GRM4-induced cell proliferation, migration and invasion.

**Conclusions:**

Our results suggest that GRM4 might be a tumor suppressor gene in breast cancer under the direct regulation of miR-328-3p and miR-370-3p.

## Background

Glutamate is one of the most important excitatory neurotransmitters in the central nervous system. Glutamate regulates the intracellular signaling pathway through binding to ionotropic glutamate receptors (iGluR) or glutamate metabotropic receptor (GRM), triggering a series of physiological and pathological effects [1]. The GRM protein family contains eight members from GRM1 to GRM8, all of which belong to G-protein-coupled receptors (GPCR). Based on the similarity of amino acid sequences and pharmacological properties, the GRM protein family could be further divided into three subgroups: group I contains GRM1 and GRM5, which couples with Gq/11; group II contains GRM2 and GRM3, which couples with Gi/o to reduce the formation of cyclic adenosine monophosphate (cAMP); group III contains GRM4, GRM6, GRM7 and GRM8, which couples with both Gi and Go. The coupling status of GRM proteins and GPCR transfers signals to secondary messengers and downstream pathways, resulting in slow physiological reactions. Therefore, the GRM family plays important roles in the regulation of ion channels, neuronal excitability and neurotransmitter release [2].

**Fig. 1:**
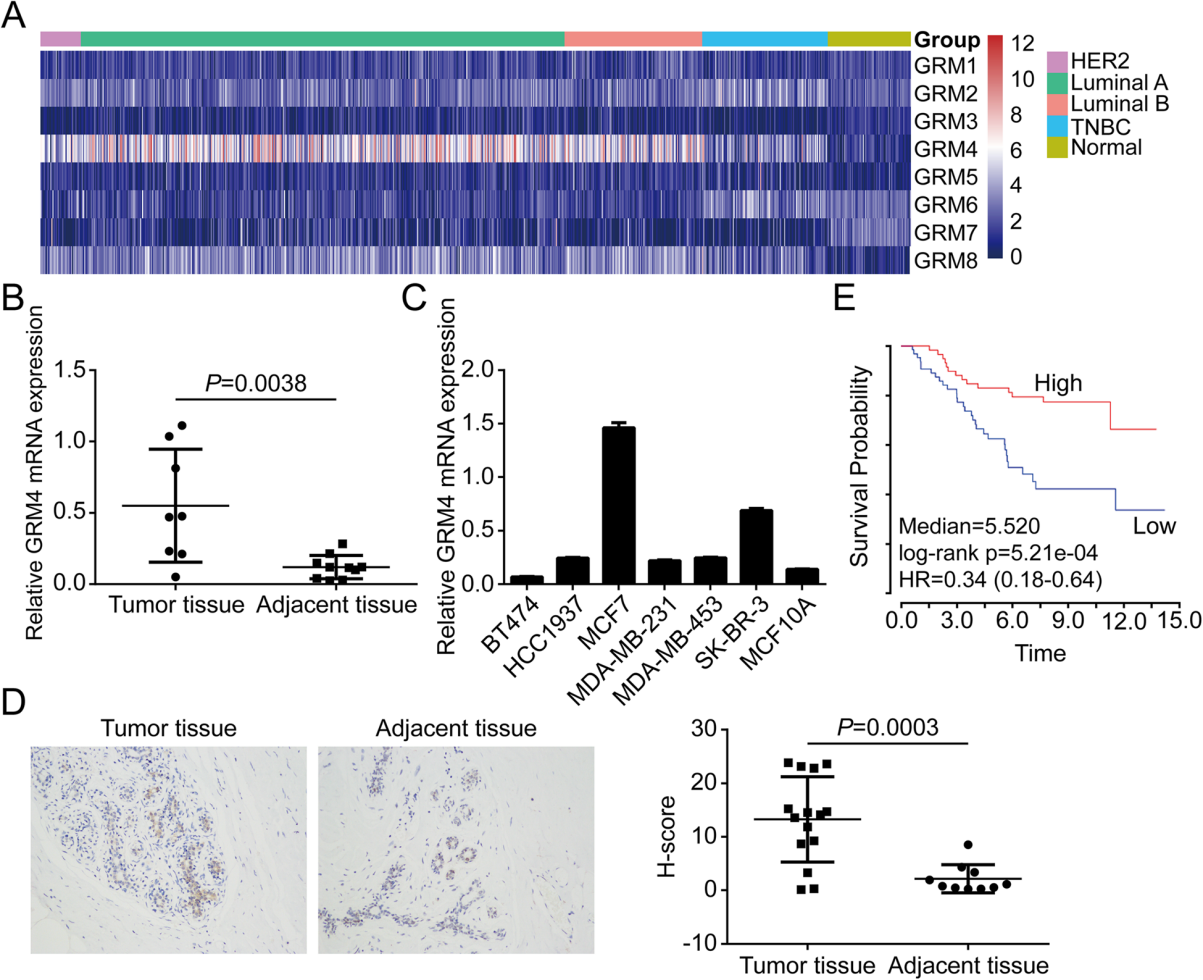
GRM4 is a potential biomarker for breast cancer. **a**. Heatmap displays the mRNA expression patterns of eight family members of GRM in four subtypes of breast cancer. **b**. The mRNA expression levels of GRM4 in 8 breast cancer tissues and 10 non-tumor tissues were measured by qPCR. **c**. GRM4 mRNA expression levels in six breast cancer cell lines and mammary epithelial cell MCF10A were measured by qPCR. **d** The protein expression levels of GRM4 in 15 breast cancer tissues and 10 control tissues were measured by immunohistochemistry (IHC). Representative images are shown on the left. **e**. Kaplan–Meier analysis compared the overall survival between breast cancer patients with high GRM4 expression and low GRM4 expression. *P*<0.001 by long rank test

Although many studies have reported the functions of GRM family in the central nervous system, their roles in the development and progression of malignancies remain largely unknown. Studies have shown that glutamate promotes the proliferation, invasion and migration abilities through binding to GRM receptors in prostatic cancer cells [3]. Among the eight GRM family members, the function of GRM5 has been investigated the most. The expression of GRM5 was up-regulated in squamous cell carcinoma and overexpression of GRM5 accelerated tumor growth [4]. GRM5, but not its group I member GRM1, was also expressed in liver tissues and enhanced the migration and invasion of hepatoma carcinoma cells through MAPK/ERK signaling [5]. However, the roles played by other GRM family members in various malignancies remain to be explored. To discover the GRM family member with significant roles in breast cancer (BC), we downloaded the RNA sequencing data of BC in TCGA database (https://gdc-portal.nci.nih.gov/) and analyzed the mRNA expression of all GRM family members. GRM4 was the only protein with a higher expression level in BC tissues (Fig. 1a). In this case, GRM4 was included in the following studies.

GRM4 was reported to be involved in adaptive immunity reactions in cancers [6]. GRM4 gene polymorphisms were also closely associated with susceptibility and clinicopathological characteristics of osteosarcoma [7–9]. We speculated that GRM4 might be important in cancers. In this study, we found that the expression of GRM4 could be detected in BC, but not in normal mammary tissue. Surprisingly, GRM4 inhibited the proliferation, invasion and migration abilities of BC cells. Moreover, we found two miRNAs, miR-328-3p and miR-370-3p, that directly targeted GRM4 and counteracted the inhibitory effect of GRM4. Our study provides a novel understanding on the role of GRM4 in BC and indicates a potential therapeutic strategy on targeting the membrane protein GRM4.

## Methods

### Cell lines and cell culture

Breast cancer cell lines BT474, HCC1937, MCF7, MDA-MB-231, MDA-MB-453, SK-BR-3 and MCF10A were purchased from the Typical Culture Preservation Commission Cell Bank (Chinese Academy of Sciences, Shanghai, China) in October, 2017. Cells were cultured in Dulbecco’s modified Eagle medium (DMEM) supplemented with 10% fetal bovine serum (ThermoFisher Scientific, Waltham, MA, USA), 100 μg/ml streptomycin, and 100 U/ml penicillin. These cell lines have been authenticated by quadruplex fluorescent short tandem repeat (STR) typing. The cells have also been tested for mycoplasma contamination using mycoplasma detection kit (Cat: CA1080, Solarbio, China), in order to confirm that cells were free from contamination before study.

### Bioinformatics analysis of GRM4 expression

The RNA sequencing V2 data of BC with corresponding clinical information were downloaded from TCGA database. The final analysis contained samples with detailed subtype information, including 37 HER2+ (HER2+, ER-, PR-) samples, 443 luminal A samples, 126 luminal B samples, 115 triple-negative BC (TNBC) samples and 76 para-carcinoma tissues. The expression patterns of eight members of GRM family, after log2 transformation, was integrated in a heat map using the R software pheatmap (Version: 1.0.8).

### Quantitative polymerase chain reaction (qPCR)

A qPCR assay was utilized to detect miR-328-3p, miR-370-3p and GRM4 expression in BC tissues and different BC cells and to measure GRM4 expression changes via regulation by various miRNA mimics and inhibitors. Total RNA was isolated using the classic Trizol method. Tissue samples were ground in liquid nitrogen and mixed with Trizol (1 ml/100 mg). Trizol-treated tissues and cells were centrifuged at 12000×g for 10 min at 4 °C and the supernatants were then mixed with chloroform. The samples were centrifuged again (12,000 x g) and the supernatants were transferred into an isopropanol-containing tube to precipitate the RNA. The precipitates were washed with 75% ethyl alcohol and resuspended in DEPC water. The cDNA synthesis and qPCR protocol were conducted using TransScript® Green One-Step qRT-PCR SuperMix (Transgen biotech, Beijing, China). The mRNA expression was calculated using the 2^-∆∆Ct^ method by normalizing to GAPDH or U6. The GRM4 fragment was amplified using the following primers: Forward primer: AGCGAATTGGGCAGGATTCA; Reverse primer: TACTTAAGCAGCTGGGTGCC. miR-328-3p mimics: CUGGCCCUCUCUGCCCUUCCGU. miR-328-3p inhibitor: ACGGAAGGGCAGAGAGGGCCAG. miR-328-3p forward primer: CGGGCCTGGCCCTCTCTGCC; miR-328-3p reverse primer: CAGCCACAAAAGAGCACAAT. miR-370-3p mimics: GCCUGCUGGGGUGGAACCUGGU. miR-370-3p inhibitor: ACCAGGUUCCACCCCAGCAGGC. miR-370-3p forward primer: GCCTGCTGGGGTGGAACCTGGT; miR-370-3p reverse primer: CTCAACTGGTGTCGTGGA. U6 forward primer: CTCGCTTCGGCAGCACA; U6 reverse primer: AACGCTTCACGAATTTGCGT.

### Immunohistochemistry (IHC) and scoring

A total of 15 BC samples and 10 adjacent normal breast tissues were obtained under surgical operation between July 2017 and April 2018. The human tissue samples were used according to the guidelines of General Hospital of Southern Theatre Command of PLA. Written informed consents were provided by all patients.

The procedure was conducted as described elsewhere. Briefly, 4-mm paraffin-embedded sections were probed with the GRM4 antibody (1:200 dilution, Cat: ab53088, Abcam, Burlingame, CA, USA). and were scanned using Pannoramic MIDI (3D HISTECH, Hungary). The images were analyzed by Quant center software. The dark brown color indicated a strong positive expression. The pale brown color corresponded to moderate positive expression and the pale-yellow color means a weak positive expression. The H-score was calculated based on the degree of positive expression and the area of each section.

### Colony formation assay

Cell formed colonies after culturing for 15–20 days. Then cells were fixed and stained with 0.5% crystal violet. The colony numbers were counted thrice by three independent laboratory technicians.

### Transwell assay for migration and invasion analysis

Transwell assays were performed as described previously. Briefly, to investigate cell migration, BC cells were seeded in the upper transwell chamber (Cat. 3422. Corning, NY, USA) filled with DMEM and 1% FBS. The lower chamber was added 600 μl DMEM with 1% FBS. After 24 h, the cells were fixed and stained with crystal violet (0.1%). The cells migrating from the upper chamber to the lower chamber were counted and the average number of six random regions were regarded as the final result. To investigate cell invasion, the BC cells were seeded into a 24-well plate (Cat. 354,480. BD Biosciences, Franklin Lakes, NJ, USA) and these other procedures were similar as above.

### Western blot

MCF7 cells transfected with different miRNAs were lysed in RIPA buffer. Protein concentration was detected were determined, loaded on 10% SDS-polyacrylamide gels and transferred to PVDF membranes. Then the PVDF membrane were incubated with anti GRM4 antibody (ab53088, Abcam) at 4 °C overnight and subsequently incubated with an HRP-conjugated anti-rabbit antibody for 1 h. The blots were visualized by ECL methods using FUJI SUPER RX film.

### Dual luciferase reporter assay

The Dual-Luciferase® Reporter Assay System (Cat: E1910, Promega, Madison, WI, USA) was used in this assay and experimental procedure was conducted as previously described. Briefly, the promoter region of GRM4 (− 200–0) was cloned and introduced into the pGL6-luc plasmid to generate pGL6-pGRM4-luc. The 17 miRNAs were cloned and introduced into the pENTER plasmid to generate 17 different miRNA-expressing recombinant plasmids. When cells reached 80% confluence, pGL6-pGRM4-luc, pENTER-miRNA and pRLTK were co-transfected into MCF7 cells. Empty vectors were served as negative controls. After 24 h, the activity of the firefly luciferase reporter was detected using Promega Glomax, and Stop Glo Buffer was then added to measure the renila luciferase activity.

## Results

### The expression of GRM4 in BC tissues and cells

The RNA sequencing data of BC tissues and adjacent non-carcinoma tissues were acquired from the TCGA database. The expression patterns of all the GRM family members in the four subtypes of BC (HER2+, Luminal A, Luminal B and TNBC) was further collected (Fig. 1a). Results suggested not all the GRM family members were expressed in normal breast tissues. However, GRM4 was the only protein with a higher expression level in BC tissues. GRM4 expression was also higher in the Luminal A and Luminal B samples than that in the HER2+ and TNBC samples. GRM4 mRNA expression was further validated in BC tissues and cells using qPCR. As shown in Fig. 1b and c, GRM4 mRNA expression was significantly higher in both BC tissues and cells compared with that in control samples. Compared with other subtypes, GRM4 mRNA expression was higher in luminal MCF7 cells, suggesting a role of GRM4 in luminal BC (Fig. 1c). IHC analysis revealed that protein expression of GRM4 was significantly higher in BC samples than that in paired non-carcinoma tissues (*P* = 0.0003) (Fig. 1d). Kaplan–Meier analysis using the mean GRM4 expression score as a cutoff point showed that GRM4 expression was remarkably correlated with the overall survival of BC patients (Fig. 1e). Taken together, these results suggested that GRM4 might play crucial roles in BC, especially in luminal BC.

### GRM4 inhibits cell proliferation, migration and invasion in BC

**Fig. 2:**
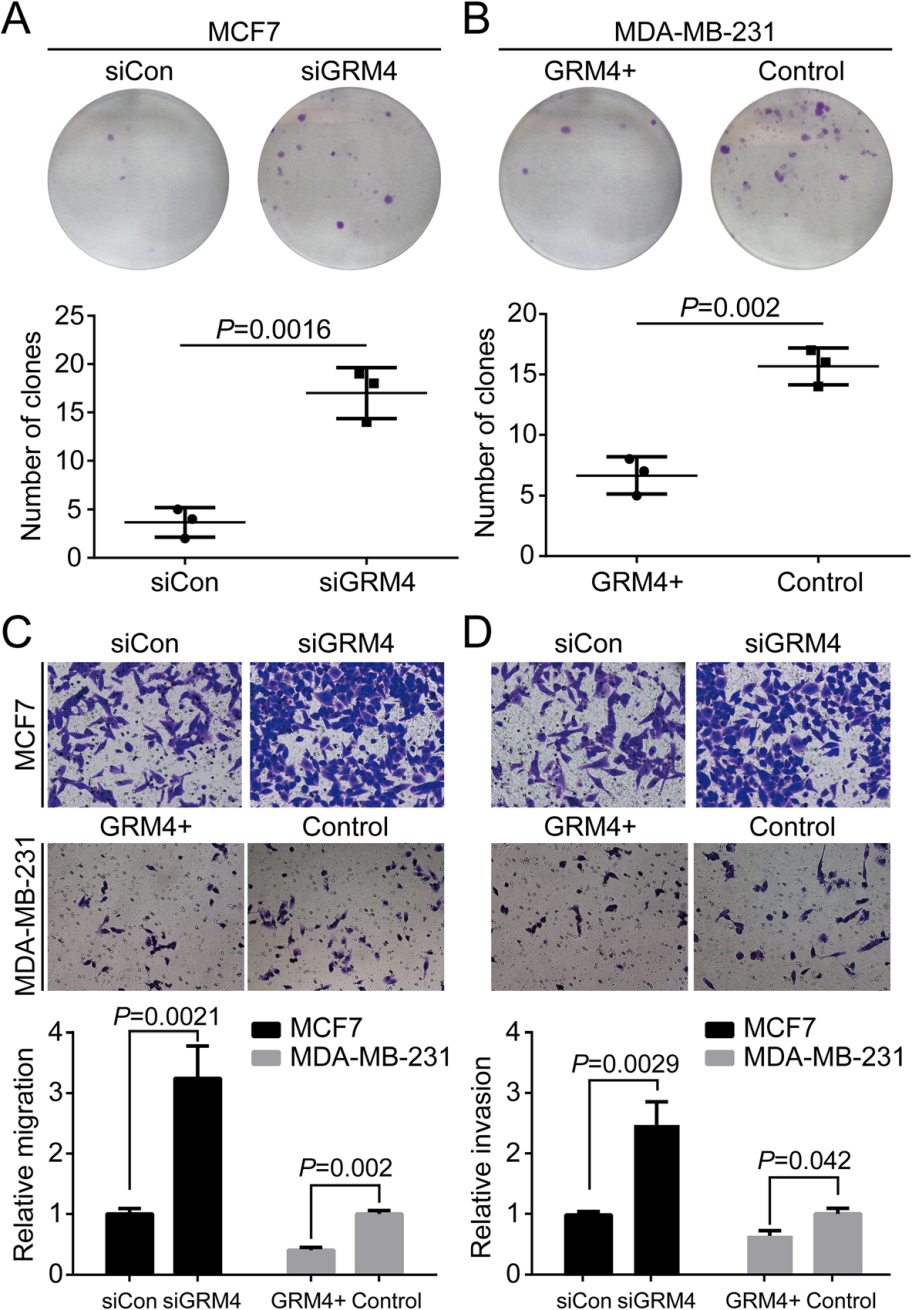
GRM4 inhibits breast cancer cell proliferation, migration and invasion. **a**. Colony formation assay measuring the number of colonies in GRM4 knock down and the control groups in MCF7. *P* = 0.0016 by One-way ANOVA. **b**. Colony formation assay measuring the number of colonies in the GRM4 knock down and the control groups in MDA-MB-231. *P* = 0.002 by One-way ANOVA. **c**. Transwell migration assay showing the number of migrated cells in MCF7 cells with GRM4 knock down or the control, and in MDA-MB-231 with GRM4 overexpression or the control. Magnification: 100×. **d**. Transwell migration assay showing the number of invaded cells in MCF7 cells with GRM4 knock down or the control, and in MDA-MB-231 with GRM4 overexpression or the control. Magnification: 100 ×

As the expression of GRM4 was increased in BC, we speculated that GRM4 might affect cell proliferation, migration and invasion of BC cells. We generated a recombinant MDA-MB-231 cell line, which stably expressed GRM4, because this cell nominally contained a low GRM4 mRNA expression level (Fig. 1c). We also knocked down GRM4 expression in MCF7 cells using siRNA. The colony formation assay showed that the cell proliferation ability was significantly enhanced by silencing GRM4, while overexpression of GRM4 inhibited colony formation in MDA-MB-231 cells (Fig. 2a and b). In the transwell migration assay, significant migration of MCF7 cells was observed when GRM4 expression was silenced (Fig. 2c). Overexpression of GRM4 in MDA-MB-231 resulted in decreased migrating cells (Fig. 2C). In the transwell invasion assay, silencing GRM4 significantly increased the number of cells that penetrated into the matrigel, while GRM4 overexpression decreased the invasion ability in MDA-MB-231. These results suggested that GRM4 inhibited in vitro cell migration and invasion in BC.

### miR-328-3p and miR-370-3p directly regulate GRM4 expression

**Fig. 3:**
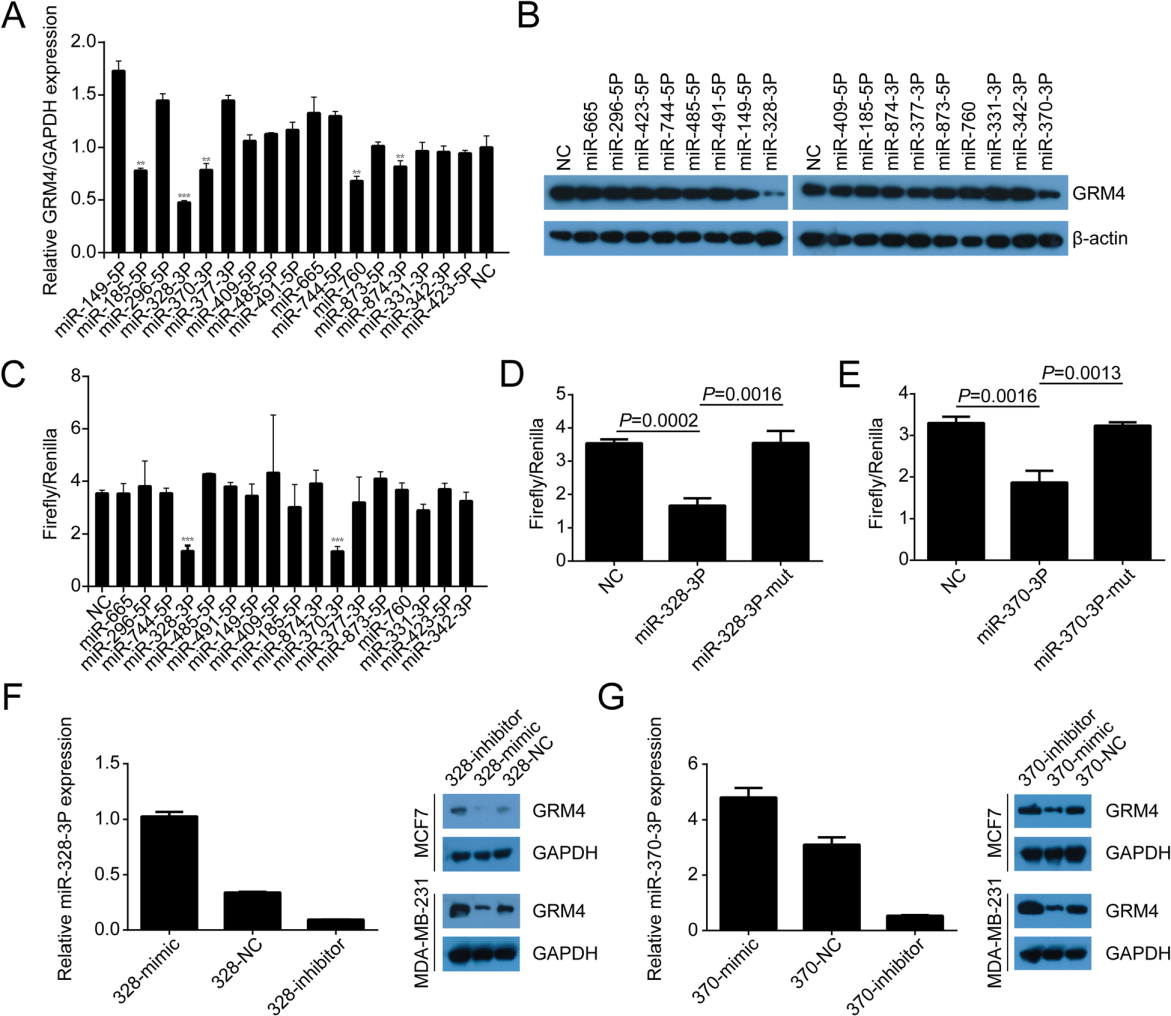
miR-328-3p and miR-370-3p directly bind to and inhibit GRM4 expression. **a**. The effect of 17 miRNAs on GRM4 expression was evaluated by qPCR. **P < 0.01 and ***P < 0.001. **b**. Western blot analysis of the effect of 17 miRNAs on GRM4 expression. **c**. Dual luciferase reporter gene assay showing the Firefly/Renilla luciferase activity after transfection of pGL6-pGRM4-luc, pRLTK and 17 pENTER-miRNA plasmids. **d**. The effect of miR-328-3p mutation on the luciferase activity suppressed by wild type miR-328-3p. **e**. The effect of miR-370-3p mutation on the luciferase activity suppressed by wild type miR-328-3p. **f** Western blot showing the effects of miR-328-3p mimic and miR-328-3p inhibitor on GRM4 expression in MCF7 and MDA-MB-231 cells. **g** Western blot showing the effects of miR-370-3p mimic and miR-370-3p inhibitor on GRM4 expression in MCF7 and MDA-MB-231 cells

miRNAs are important regulators of gene expression at the mRNA level. To explore further why the expression of GRM4 is not detected in normal breast tissue, but is up-regulated in BC, we screened miRNAs that possibly regulated GRM4 expression using the Targetscan database (http://www.targetscan.org). 17 miRNAs were selected for further investigation according to the sequence matching of these miRNAs with the 3^′^ UTR of GRM4. Each of the 17 miRNAs and the negative control vector were transfected into MCF7cellsand GRM4 mRNA expression was detected by qPCR. Compared with the negative control, five miRNAs, including miR-185-5p, miR-328-3p, miR-370-3p, miR-760 and miR-874-3p, significantly inhibited GRM4 mRNA expression (Fig. 3a). This result was confirmed by western blot. As shown in Fig. 3b, miR-328-3p and miR-370-3p strongly inhibited GRM4 protein levels, but miR-185-5p, miR-760 and miR-874-3p showed no impact on GRM4 expression. Dual luciferase reporter assays showed that miR-328-3p and miR-370-3p decreased the Firefly/Renilla activity driven by the GRM4 promoter (Fig. 3c). We generated the GRM4-binding domain deficient vector for miR-328-3p (from UGCCUUCCCGUCUCUCCCGGUC to UGCCUUGGGCUCUCAGGGCCAC) and miR-370-3p (from UGGUCCAAGGUGGGGUCGUCCG to UGGUCCAAGGUGGGCAGCAGGG). The miR-328-3p and miR-370-3p mutation vectors lost the inhibitory effect on Firefly/Renilla activity (Fig. 3d and e). To further verify the effect of miR-328-3p on GRM4 expression, miR-328-3p mimic and miR-328-3p inhibitor were transfected into MCF7 and MDA-MB-231 respectively (Fig. 3f, left). Compared with the negative control (NC) group, miR-328-3p mimic decreased the expression of GRM4 and miR-328-3p inhibitor enhanced GRM4 expression (Fig. 3F, right). Similar effects on GRM4 expression could be observed by transfecting miR-370-3p mimic and miR-370-3p inhibitor in MCF7 and MDA-MB-231 cells (Fig. 3g). These results indicated that miR-328-3p and miR-370-3p directly inhibited GRM4 expression by binding to the 3^′^ UTR of GRM4.

### The expression of miR-328-3p and miR-370-3p were down-regulated in BC but not correlated with GRM4

**Fig. 4:**
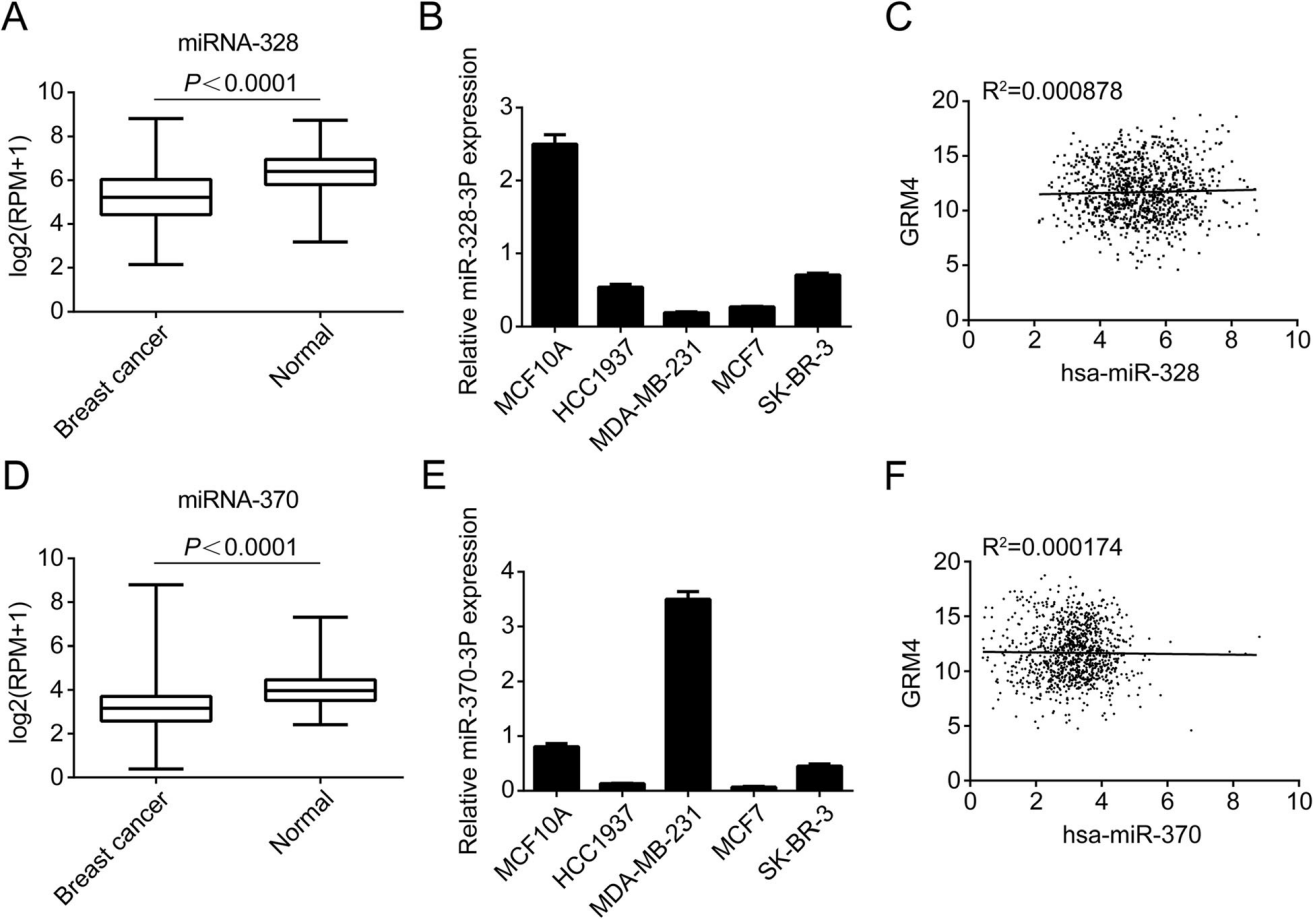
The expression levels of miRNA-328 and miRNA-370 in breast cancer tissues and cell lines and their correlation with GRM4. **a** + **d**. The expression levels of miRNA-328 (**a**) and miRNA-370 (**d**) between breast cancer and normal tissue in TCGA data set. *P*<0.0001 by One-way ANOVA. **b** + **e**. The expression levels of miRNA-328 (**b**) and miRNA-370 (**e**) in five breast cancer cell lines detected by qRT-PCR. **c** + **f**. The correlation between GRM4 and miRNA-328 (**c**) or miRNA-370 (**f**) using TCGA data set. The Pearson correlation analysis was performed

To assess whether miRNA-328-3p and miRNA-370-3p were down-regulated in BC, we explored TCGA dataset to detect the level of these two miRNAs in BC tissues and in normal tissues. As shown in Fig. 4a and Fig. 4d, the expression of miRNA-328-3p and miRNA-370-3p were effectively lower in BC compared with normal tissues (*P* < 0.0001). Next, we determined the expression level of miRNA-328-3p and miRNA-370-3p in BC cells. We used qRT-PCR to detect their expression levels in five BC cell lines. The expression of miRNA-328-3p is higher in normal breast cells MCF10A and has the lowest expression in MDA-MB-231 cell lines (Fig. 4b). However, miRNA-370-3p has the highest level in MDA-MB-231 than other four cell lines (Fig. 4e). Given that our previous data showed these two miRNAs could negatively regulate the expression of GRM4, it was of interest to see if the same regulation was manifest in another BC cohort. To this end, we explored the correlation of miRNA-328, miRNA-370 and GRM4 using TCGA published data sets. However, in this manner, it was determined that the expression level of both two miRNAs don’t correlate with GRM4 expression (Fig. 4c and f).

### miR-328-3p and miR-370-3p mediate BC cell proliferation, migration and invasion induced by GRM4

**Fig. 5:**
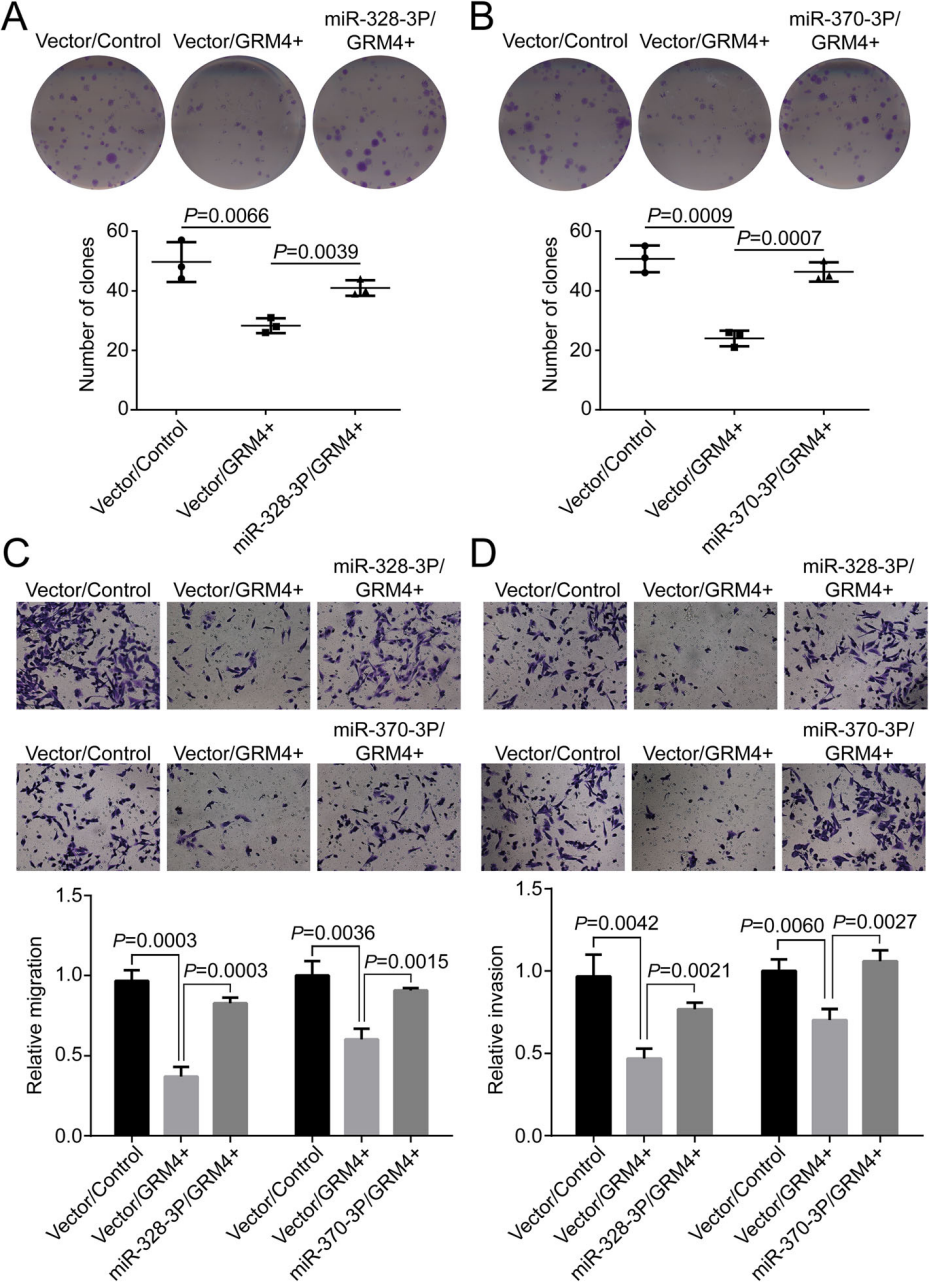
miR-328-3p and miR-370-3p counteract the inhibitory effect of GRM4 on cell proliferation, migration and invasion. **a**. miR-328-3p reverses the GRM4-induced decrease in colony formation ability in MDA-MB-231. **b**. miR-370-3p enhanced colony formation was inhibited by GRM4 overexpression in MDA-MB-231. **c**. Transwell migration assay measuring the effects of miR-328-3p and miR-370-3p on the motility of GRM4 overexpressing MDA-MB-231 cells. **d**. Transwell invasion assay measuring the effects of miR-328-3p and miR-370-3p on the motility of GRM4 overexpressing MDA-MB-231 cells

To our knowledge, the roles of miR-328-3p and miR-370-3p in BC remain unclear. To verify whether miR-328-3p and miR-370-3p could counteract the GRM4-induced inhibitory effect on BC cell proliferation, migration and invasion, miR-328-3p and miR-370-3p were transfected into MDA-MB-231, which stably expressed GRM4. The expression of miR-328-3p and miR-370-3p led to an increased number of colonies of GRM4 overexpressing cells compared with the control cells (Fig. 5a and b). In the transwell migration assay, miR-328-3p and miR-370-3p counteracted the GRM4-dependent reduction of migrating cells (Fig. 5c). miR-328-3p and miR-370-3p also promoted the invasive capacity of GRM4 overexpressing cells in the transwell invasion assay (Fig. 5d). Based on these results, we concluded that miR-328-3p and miR-370-3p negatively regulated the expression of GRM4 and inhibited GRM4-mediated cell proliferation, migration and invasion.

## Discussion

Proteins of the GRM family have long been known to be important in the transmission of neural signals, participating in various cerebral activities, such as the regulation of nerve cells, axon development and synaptic plasticity formation. GRM proteins couple with G-proteins through their intracellular regions to deliver molecular signals, resulting in metabolic changes in nerve cells. Although the functions of GRM proteins in the central nervous system have been reported abundantly, their roles in tumor malignancies, especially in BC, remain unclear. Previous studies have provided some clues for this study design. PET imaging showed that glutamine is avidly taken up by gliomas, and can be used to assess in vivo metabolic nutrient uptake in gliomas [10]. This phenomenon may relate to the up-regulation of GRM proteins in gliomas [11]. Glutamate release promotes the growth and metastasis of malignant gliomas and melanoma through GRM5 and the downstream PI3k/Akt pathway [12, 13]. Our results found that the expression of GRM4 is increased in BC and is associated with better prognoses of patients. GRM4 functions as a tumor suppressor that inhibits cancer cell proliferation, migration and invasion. We also found that miR-328-3p and miR-370-3p bound directly to the 3^′^ UTR of GRM4, inhibit GRM4 expression and its biological functions. These results might partly explain why GRM4 mRNA expression is increased in BC. Although the expression of miR-328-3p and miR-370-3p was down-regulated in BC tissues and cells (Fig. 4a, b, d and e), no correlations were found between the expression of miR-328-3p and GRM4, as well as the expression of miR-370-3p and GRM4 (Fig. 4c and f). This might be due to the inconsistence expression levels of GRM4 between different subtypes of BC. Whether the negative regulation of miR-328-3p and miR-370-3p on GRM4 expression existed in specific subtypes needs further characterization.

miR-328 has been considered as a tumor suppressor in esophageal cancer [14], non-small cell lung cancer [15], TNBC [16] and osteosarcomas [17], but might also be considered as an oncogene in invasive breast carcinoma [18]. In esophageal cancer, miR-328 suppresses cell survival by targeting PLCE1. In A549 lung cancer cells, increased expression of miR-328-3p was found to inhibit cell survival and restore lung cell sensitivity to radiotherapy [14]. The TNBC cell motility was found significantly decreased after transfecting miR-328-3p mimics [16]. Our results suggested miR-328-3 inhibited the expression of GRM4, a tumor suppressor in MCF7 luminal BC cells and overexpression of miR-328-3p promoted proliferation, migration and invasion in GRM4 stably expressing cells, indicating that miR-328-3p may switch to an oncogene in the context of GRM4.

miR-370 plays dual roles in several types of cancers. As an oncogene, higher expression of miR-370 corresponded to enhanced melanoma cell proliferation and invasion and decreased cell apoptosis by targeting pyruvate dehydrogenase B [19]. miR-370 also enhances cell proliferation and migration in gastric cancer by targeting EGFR [20]. As a tumor suppressor, expression of miR-370 was decreased in thyroid cancer and suppressed progression and inhibited the Wnt signaling pathway in thyroid cancer [21]. miR-370 functions as a sponge of hsa_circ_0061140 in ovarian cancer and inhibits hsa_circ_0061140-induced cell growth and metastasis [22]. Our results indicated that miR-370 targeted GRM4 and reversed GRM4-mediated cell proliferation and migration. This result is consistent with the report that upregulation of miR-370 in BC is correlated with lymph node metastasis, advanced stage, frequent perineural invasion and poor disease-free survival [23].

## Conclusions

In conclusion, this study suggested that GRM4 might have a potential to serve as a biomarker for the clinical diagnosis of BC by detecting its mRNA or protein levels using immunohistochemistry. Future studies should focus on the molecular mechanisms underlying GRM4-mediated inhibition of BC proliferation and on developing novel clinic strategies by targeting GRM4.

## Data Availability

The dataset supporting the conclusions of this article is included within the article.

## Abbreviations

cAMP: cyclic adenosine monophosphate
DMEM: Dulbecco’s modified Eagle medium
GPCR: G-protein-coupled receptors
GRM: Glutamate metabotropic receptors
iGluR: ionotropic glutamate receptors
qPCR: Quantitative polymerase chain reaction
TNBC: Triple-negative breast cancer

## References

[1] Elegheert J, Kakegawa W, Clay JE, Shanks NF, Behiels E, Matsuda K, Kohda K, Miura E, Rossmann M, Mitakidis N (2016). Structural basis for integration of GluD receptors within synaptic organizer complexes. SCIENCE.

[2] Ramos C, Chardonnet S, Marchand CH, Decottignies P, Ango F, Daniel H, Le Marechal P (2012). Native presynaptic metabotropic glutamate receptor 4 (mGluR4) interacts with exocytosis proteins in rat cerebellum. J Biol Chem.

[3] Koochekpour S, Majumdar S, Azabdaftari G, Attwood K, Scioneaux R, Subramani D, Manhardt C, Lorusso GD, Willard SS, Thompson H (2012). Serum glutamate levels correlate with Gleason score and glutamate blockade decreases proliferation, migration, and invasion and induces apoptosis in prostate cancer cells. Clin Cancer Res.

[4] Park SY, Lee SA, Han IH, Yoo BC, Lee SH, Park JY, Cha IH, Kim J, Choi SW (2007). Clinical significance of metabotropic glutamate receptor 5 expression in oral squamous cell carcinoma. Oncol Rep.

[5] Wu YL, Wang NN, Gu L, Yang HM, Xia N, Zhang H (2012). The suppressive effect of metabotropic glutamate receptor 5 (mGlu5) inhibition on hepatocarcinogenesis. BIOCHIMIE.

[6] Fallarino F, Volpi C, Fazio F, Notartomaso S, Vacca C, Busceti C, Bicciato S, Battaglia G, Bruno V, Puccetti P (2010). Metabotropic glutamate receptor-4 modulates adaptive immunity and restrains neuroinflammation. Nat Med.

[7] Savage SA, Mirabello L, Wang Z, Gastier-Foster JM, Gorlick R, Khanna C, Flanagan AM, Tirabosco R, Andrulis IL, Wunder JS (2013). Genome-wide association study identifies two susceptibility loci for osteosarcoma. Nat Genet.

[8] Jiang C, Chen H, Shao L, Dong Y (2014). GRM4 gene polymorphism is associated with susceptibility and prognosis of osteosarcoma in a Chinese Han population. Med Oncol.

[9] Wang K, Zhao J, He M, Fowdur M, Jiang T, Luo S (2016). Association of GRM4 gene polymorphisms with susceptibility and clinicopathological characteristics of osteosarcoma in Guangxi Chinese population. Tumour Biol.

[10] Venneti S, Dunphy MP, Zhang H, Pitter KL, Zanzonico P, Campos C, Carlin SD, La Rocca G, Lyashchenko S, Ploessl K (2015). Glutamine-based PET imaging facilitates enhanced metabolic evaluation of gliomas in vivo. SCI TRANSL MED.

[11] Prickett TD, Samuels Y (2012). Molecular pathways: dysregulated glutamatergic signaling pathways in cancer. Clin Cancer Res.

[12] Takano T, Lin JH, Arcuino G, Gao Q, Yang J, Nedergaard M (2001). Glutamate release promotes growth of malignant gliomas. Nat Med.

[13] Choi KY, Chang K, Pickel JM, Badger JN, Roche KW (2011). Expression of the metabotropic glutamate receptor 5 (mGluR5) induces melanoma in transgenic mice. Proc Natl Acad Sci U S A.

[14] Han N, Zhao W, Zhang Z, Zheng P (2016). MiR-328 suppresses the survival of esophageal cancer cells by targeting PLCE1. Biochem Biophys Res Commun.

[15] Ma W, Ma CN, Zhou NN, Li XD, Zhang YJ (2016). Up- regulation of miR-328-3P sensitizes non-small cell lung cancer to radiotherapy. Sci Rep.

[16] Al-Othman N, Hammad H, Ahram M (2018). Dihydrotestosterone regulates expression of CD44 via miR-328-3P in triple-negative breast cancer cells. GENE.

[17] Yang SF, Lee WJ, Tan P, Tang CH, Hsiao M, Hsieh FK, Chien MH (2015). Upregulation of miR-328 and inhibition of CREB-DNA-binding activity are critical for resveratrol-mediated suppression of matrix metalloproteinase-2 and subsequent metastatic ability in human osteosarcomas. ONCOTARGET.

[18] Saberi A, Danyaei A, Neisi N, Dastoorpoor M, Tahmasbi BM (2016). MiR-328 may be considered as an oncogene in human invasive breast carcinoma. Iran Red Crescent Med J.

[19] Wei S, Ma W (2017). MiR-370 functions as oncogene in melanoma by direct targeting pyruvate dehydrogenase B. Biomed Pharmacother.

[20] Ning T, Zhang H, Wang X, Li S, Zhang L, Deng T, Zhou L, Liu R, Wang X, Bai M (2017). miR-370 regulates cell proliferation and migration by targeting EGFR in gastric cancer. Oncol Rep.

[21] Chen F, Feng Z, Zhu J, Liu P, Yang C, Huang R, Deng Z. Emerging roles of circRNA_NEK6 targeting miR-370-3P in the proliferation and invasion of thyroid cancer via Wnt signaling pathway. CANCER BIOL THER. 2018:1–14. 10.1080/15384047.2018.1480888 PMC6301817 30207869

[22] Chen Q, Zhang J, He Y, Wang Y (2018). hsa_circ_0061140 knockdown reverses FOXM1-mediated cell growth and metastasis in ovarian Cancer through miR-370 sponge activity. Mol Ther Nucleic Acids.

[23] Sim J, Ahn H, Abdul R, Kim H, Yi KJ, Chung YM, Chung MS, Paik SS, Song YS, Jang K (2015). High MicroRNA-370 expression correlates with tumor progression and poor prognosis in breast Cancer. J Breast Cancer.

